# Lower Ventromedial Prefrontal Cortex Glutamate Levels in Patients With Obsessive–Compulsive Disorder

**DOI:** 10.3389/fpsyt.2021.668304

**Published:** 2021-06-08

**Authors:** Marcelo C. Batistuzzo, Bruna A. Sottili, Roseli G. Shavitt, Antonio C. Lopes, Carolina Cappi, Maria Alice de Mathis, Bruno Pastorello, Juliana B. Diniz, Renata M. F. Silva, Euripedes C. Miguel, Marcelo Q. Hoexter, Maria C. Otaduy

**Affiliations:** ^1^Department & Institute of Psychiatry, University of São Paulo Medical School, São Paulo, Brazil; ^2^Department of Methods and Techniques in Psychology, Pontifical Catholic University, São Paulo, Brazil; ^3^Laboratory of Magnetic Resonance (LIM44), Department and Institute of Radiology, University of São Paulo (InRad-FMUSP), São Paulo, Brazil; ^4^Laboratório Interdisciplinar de Neurociências Clínicas (LiNC), Universidade Federal de São Paulo (UNIFESP), São Paulo, Brazil

**Keywords:** obsessive-compulsive disorder, magnetic resonance spectroscopy, prefrontal cortex, neurometabolic alterations, Glutamate, GABA

## Abstract

**Background:** Recent studies using magnetic resonance spectroscopy (^1^H-MRS) indicate that patients with obsessive–compulsive disorder (OCD) present abnormal levels of glutamate (Glu) and gamma aminobutyric acid (GABA) in the frontal and striatal regions of the brain. These abnormalities could be related to the hyperactivation observed in cortico-striatal circuits of patients with OCD. However, most of the previous ^1^H-MRS studies were not capable of differentiating the signal from metabolites that overlap in the spectrum, such as Glu and glutamine (Gln), and referred to the detected signal as the composite measure—Glx (sum of Glu and Gln). In this study, we used a two-dimensional JPRESS ^1^H-MRS sequence that allows the discrimination of overlapping metabolites by observing the differences in J-coupling, leading to higher accuracy in the quantification of all metabolites. Our objective was to identify possible alterations in the neurometabolism of OCD, focusing on Glu and GABA, which are key neurotransmitters in the brain that could provide insights into the underlying neurochemistry of a putative excitatory/inhibitory imbalance. Secondary analysis was performed including metabolites such as Gln, creatine (Cr), N-acetylaspartate, glutathione, choline, lactate, and myo-inositol.

**Methods:** Fifty-nine patients with OCD and 42 healthy controls (HCs) underwent 3T ^1^H-MRS in the ventromedial prefrontal cortex (vmPFC, 30 × 25 × 25 mm^3^). Metabolites were quantified using ProFit (version 2.0) and Cr as a reference. Furthermore, Glu/GABA and Glu/Gln ratios were calculated. Generalized linear models (GLMs) were conducted using each metabolite as a dependent variable and age, sex, and gray matter fraction (fGM) as confounding factors. GLM analysis was also used to test for associations between clinical symptoms and neurometabolites.

**Results:** The GLM analysis indicated lower levels of Glu/Cr in patients with OCD (*z* = 2.540; *p* = 0.011). No other comparisons reached significant differences between groups for all the metabolites studied. No associations between metabolites and clinical symptoms were detected.

**Conclusions:** The decreased Glu/Cr concentrations in the vmPFC of patients with OCD indicate a neurochemical imbalance in the excitatory neurotransmission that could be associated with the neurobiology of the disease and may be relevant for the pathophysiology of OCD.

## Introduction

Obsessive–compulsive disorder (OCD) is a psychiatric disease that affects 1–4% of the population (lifetime) around the world (1, 2). Although its pathophysiology remains not entirely understood, there is a consensus that OCD is characterized by abnormalities in the cortico-striato-thalamo-cortical (CSTC) circuitry. In the last decade, studies using proton magnetic resonance spectroscopy (^1^H-MRS), the only technique that allows to non-invasively estimate the levels of brain neurochemicals *in vivo*, showed that patients with OCD could present altered glutamatergic (excitatory) and GABAergic (inhibitory) neurotransmission in the prefrontal cortex and striatal brain regions (3–8). However, inconclusive findings in the literature indicate that there is still a need for a thorough investigation (9).

The role of glutamate (Glu) signaling in the treatment of OCD has been investigated in therapeutic clinical trials in the literature (10–14), including positive randomized clinical trials that used glutamatergic agents as the main outcome or as an enhancer (15–18). On the other hand, augmentation studies with glutamatergic agents in patients with OCD did not show superiority to the simple administration of selective serotonin reuptake inhibitors (19, 20). In addition, candidate gene studies showed the involvement of genes coding for the glutamate signaling cascade, especially the DLGAP/SAPAP family genes (21, 22). Overall, these studies advanced the field by adding information on the neurobiological model of OCD, by testing modulation effects or associated genes, but they did not measure glutamate directly. Therefore, neurobiological research and, more specifically, *in vivo* neurochemical research on patients with OCD, is important to elucidate the glutamatergic hypothesis in OCD (11) and the role of other metabolites as well.

The most prominent neurobiological model of OCD involves abnormalities (typically hyperactivation) in the multiple and parallel CSTC circuits (22, 23). Generally, the role of gamma aminobutyric acid (GABA) in these circuits has been relatively understudied, but one hypothesis is that diminished levels of this metabolite in the prefrontal cortex (PFC) would be one of the reasons for the striatal dopaminergic and glutamatergic hyperactivity observed in patients with OCD (24). In this sense, two possible GABA paths are postulated: a direct path, in which GABA projections from the ventromedial PFC (vmPFC) reach the striatum, and a second one, indirectly, via projections to the orbitofrontal cortex.

Brain circuits related to fear and reward that encompass the vmPFC are relevant for the neurobiology of OCD and have been previously associated with the disorder both in theory and practice (25, 26). Structural, but mainly functional, abnormalities within the vmPFC were detected in imaging studies and could be related to alterations in the CSTC circuits described in the neuroimaging literature (22, 27). The vmPFC is thought to be involved with the “affective system,” participating, for example, in affective behaviors as processing affects or rewards (26). Of note, this region has been shown to be crucial to the retention of extinction learning in fear conditioning paradigms. As the habituation promoted by one of the main psychotherapeutic treatments of OCD (cognitive behavioral therapy, with exposure, and response prevention techniques) is somehow similar to extinction as evaluated in fear conditioning paradigms, some authors have proposed that proper activation of the vmPFC could be implicated in the treatment response to psychotherapy among OCD patients (28). Therefore, due to its importance in the neurobiological model of OCD, we investigated the vmPFC in the present study.

^1^H-MRS studies evaluating Glu concentrations in the brain of patients with OCD have found mixed results. Some of them show a reduction in Glu concentration in patients when compared to controls (29) while others have reported increased Glx (Glu + glutamine) levels (30). However, most of the findings did not report any differences (4, 6, 8, 9, 31). Therefore, despite the growing literature in the OCD ^1^H-MRS field in recent years (14, 32), there is still no consensus on the role of the glutamatergic cycle in OCD. Regarding GABA, this is a much more difficult metabolite to detect, as it requires specific ^1^H-MRS editing techniques. In a recent review of the literature that included only ^1^H-MRS studies that used scan fields strength of 3 T, the authors concluded that most studies did not demonstrate any neurometabolic abnormalities in OCD patients when compared to healthy controls, although they indicated that altered GABA levels in the rostral anterior cingulate cortex (rACC) is one of the most consistent findings (9). Still, while they reported two studies showing lower GABA concentrations in rACC in adults with OCD (4, 6), a more recent study has found higher concentrations of this metabolite in patients with OCD in the ACC (7). Therefore, the current evidence is not sufficiently strong for elucidating the role of neurometabolites in OCD, particularly Glu and GABA, and further research is needed in this field.

Factors that can contribute to results heterogeneity among different studies include voxel size and anatomical placement and the inherent variability of the OCD population. Moreover, since conventional ^1^H-MRS is not ideal to detect Glu, glutamine (Gln), and GABA due to their low signal and partial overlap with other metabolites, this could be considered a limitation of the previous studies, since most of them were incapable of disentangling the specific signal from each metabolite. Hence, this study used a two-dimensional JPRESS ^1^H-MRS sequence that allows the discrimination of overlapping metabolites by observing the differences in J-coupling (second dimension of the spectrum), leading to higher accuracy of all metabolites in the brain, including Glu, Gln, and GABA (33, 34).

The main objective of this study was to quantify specific metabolites (especially Glu/Cr and GABA/Cr) in the vmPFC of OCD patients and to compare them with HC. In our statistical model, we considered demographic data (such as age and sex) and also the fraction of gray matter (fGM) in the MRS voxel, aiming to control for possible confounders. Additionally, since alterations in Glu/Cr levels could be accompanied by corresponding GABA/Cr alterations [for example, see Scotti-Muzzi (35), which used the same ^1^H-MRS technique to acquire the data], an imbalance in the Glu/GABA ratio between groups could also add information regarding the neurochemistry of OCD. Thus, we also explored the Glu/GABA ratio, which has not been systematically studied previously in the OCD field. We hypothesized that the current investigation could improve our comprehension of prior results with mixed findings, by studying a large sample of OCD patients with a ^1^H-MRS sequence more specific for overlapping metabolites.

## Materials and Methods

### Ethical Issues

This project was approved by the Ethics Committee for Analysis of Research Projects (CAPPesq) at Faculdade de Medicina da Universidade de São Paulo (FMUSP). All participants signed a written informed consent after a thorough description of the study and the assurance that their decision to participate would not interfere with their access to treatment. Participants received financial compensation for transportation and refreshments during the study.

### Participants and Inclusion and Exclusion Criteria

Participants were recruited from 2014 to 2017, during a period of 3 years and 1 month. Inclusion criteria for OCD patients were the following: (a) age between 18 and 65 years; (b) primary diagnosis of OCD according to the Diagnostic and Statistical Manual of Mental Disorders, fourth edition (DSM-IV), confirmed by the Structured Clinical Interview for DSM-IV Axis I disorders (SCID-I); (c) Y-BOCS score ≥16 or ≥10 only for only obsessions or compulsions; and (d) to be on a stable medication regimen for the last 6 weeks or off medication. Exclusion criteria were the following: (a) IQ below 80; (b) comorbidity with schizophrenia or bipolar disorder; (c) any contraindication to MRI, such as pacemakers or cochlear implant, etc.; (d) claustrophobia or not being able to tolerate the exam; (e) past or current substance abuse or dependence; and (f) head trauma with loss of consciousness. HC had no history of physical or psychiatric disorders on the basis of the SCID interview and had to meet the same inclusion/exclusion criteria, with the exception of the OCD-related ones.

### Clinical Measures

All participants were interviewed by experienced clinical psychologists and completed the same battery of clinical assessments with the standardized instruments described below. All interviews were conducted in person and lasted ~2 h. To evaluate psychiatric disorders, the SCID-I (axis I) and an additional module for impulse-control disorders according to DSM-IV criteria were used for all participants (36). The OCD diagnosis was established by clinicians with long experience in OCD assessment and treatment (ACL, AM, JBD, RMFS, and RGS). The present Portuguese version of the SCID showed good interrater reliability (37).

OCD severity was measured using the Y-BOCS severity scale (38), which is a 10-item semistructured clinician-administered measure of obsession and compulsion severity. Each item can score from 0 to 4, with a maximum of 40 (20 for obsessions and 20 for compulsions). The Y-BOCS has been considered the gold-standard instrument for the assessment of OCD symptoms (38), with good psychometric properties including Brazilian samples evaluated in Portuguese (39).

Finally, the 21-item Beck Depression Inventory (BDI) (40) measures cognitive, behavioral, and somatic symptoms associated with depression. The Beck Anxiety Inventory (BAI) is a 21-question multiple choice, each answer being scored on a scale value of 0 (not at all) to 3 (severely), used for measuring the severity of anxiety in adults (41). Both scales were applied to all participants, they are consolidated scales that were translated into Brazilian Portuguese and validated in our environment previously (42).

### Procedures

#### Image Acquisition

**Figure 1:**
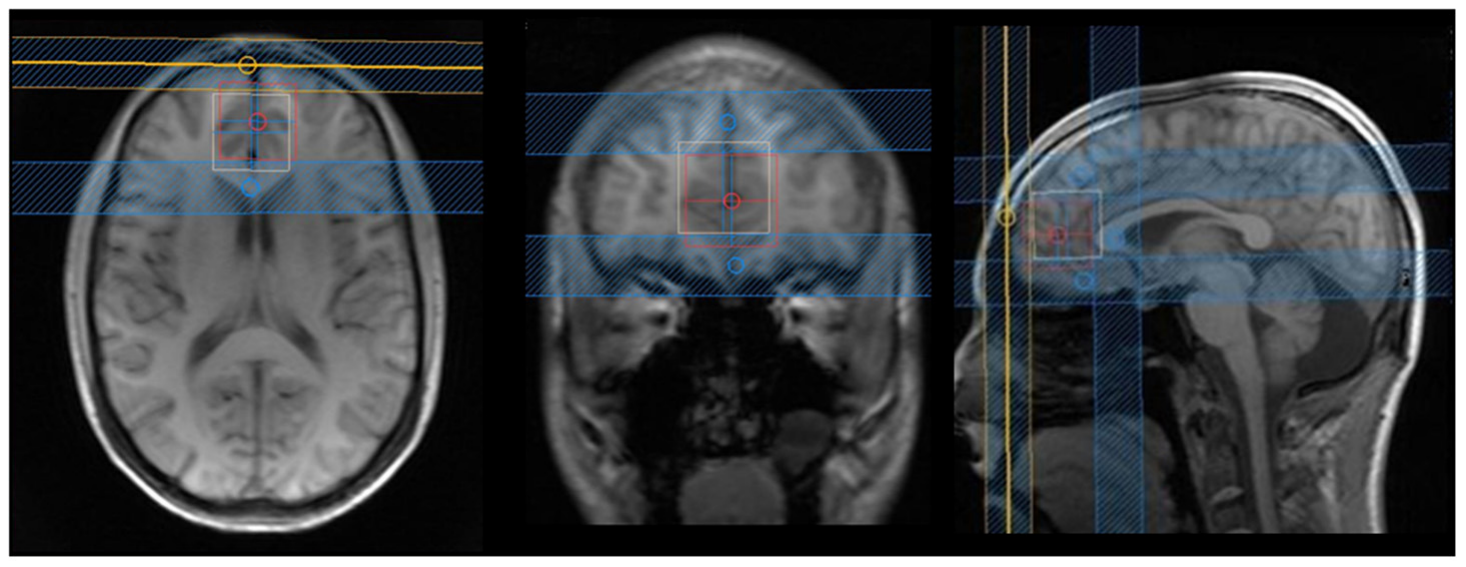
Axial, coronal, and sagittal images from the voxel position in the ventromedial prefrontal cortex: blue lines are suppression bands, used in the Y- and Z-axes. We analyzed that only the voxel that was the overlap between the white and red squares in order to reduce effects of chemical shift voxel displacement. The following rationale was used: the bottom portion of the voxel was aligned with the anterior–posterior commissure; in the anterior/posterior axis, the corpus callosum was used as reference (just ahead of the genu of corpus callosum); and in the lateral axis, the voxel was situated in the most medial portion of the brain.

The entire MRI scan lasted ~1 h, and the images were acquired on a Philips 3 T Achieva scanner (Philips Healthcare, Best, The Netherlands) using a 32-channel head coil. Spectroscopy was acquired with a two-dimensional JPRESS ^1^H-MRS sequence (33), a technique based on a conventional PRESS spin echo (single voxel) that varies the echo time of the acquisition, encoding the J coupling evolution in an additional dimension. In other words, the ^1^H-MRS signal is measured not only as a function of the chemical shift (expressed by the Larmor frequency, as in conventional one-dimensional spectroscopy) but also as a function of the coupling constant J in Hz. With the coupling constant J, it is possible to resolve the signals from overlapping multiplets, such as Glu and Gln. This sequence lasted ~25 min and was obtained with the following parameters: the voxel was positioned in the ventromedial PFC (Figure 1) with a size of 30 mm (L–R) × 25 mm (I–S) × 25 mm (A–P); minimum echo time (TE) used was 31 ms, and TE was incremented in 100 steps of 2 ms each; for every time increment ΔTE, the maximum-echo sampling started the acquisition ΔTE/2 earlier with respect to the echo top, the repetition time (TR) was 1,600 ms, and 8 averages were acquired for each TE step. One non-water suppressed spectrum was also acquired at each TE. The number of points per spectrum was 1,024, and the spectral bandwidth was 2,000 Hz. An automatic second-order B0 shimming routine was used, and water suppression was achieved by VAPOR (43). Additionally, in order to reduce the effect of chemical shift voxel displacement, four suppression bands were positioned at the voxel edges along the Y- and Z-axes, which present a more pronounced chemical shift artifact due to the use of 180-slice selective pulses (see Figure 1).

#### Data Processing

Metabolite quantification was obtained using Prior Knowledge Fitting (ProFit) version 2.0 running on Matlab R2011b (44). ProFit (34) works as an extension of LCModel (45) principles to fit 2D data sets. Fuchs et al. (44) improved the quantification program (ProFit, version 2.0) by introducing an experimentally acquired 2D macromolecular baseline into the fitting model and allowing for a more accurate and precise fit by accounting for the actual line shape and additional baseline distortions by self-deconvolution and spline modeling approach. An example of a ^1^H-MRS JPRESS spectrum can be found in the supplementary files (Supplementary Figure 1).

The metabolite basis set used by ProFit includes spectra from a total of 18 brain metabolites. The metabolites of interest that were analyzed in this study were Glu, GABA, Gln, N-acetylaspartate (NAA), creatine (Cr), glutathione (GSH), choline (Cho), lactate (Lac), and myo-inositol (ml). The first two metabolites, and the ratio among them (Glu/GABA), were the main objective of this study. All the other metabolites described above were also extracted and quantified but analyzed as secondary outcomes. Basis set metabolite spectra were calculated with the GAMMA library (46) using the chemical shift and J-coupling values from the literature (47, 48). Quantitative results in ProFit are given in the form of ratios to Cr signal (met/Cr). These ratios are already corrected for T2 relaxation effects since ProFit automatically calculates T2 relaxation times for each metabolite from the signal obtained at the different TEs.

To determine the brain tissue composition contained in the MRS voxel of interest, three-dimensional volumetric T1 images were obtained using the 3D turbo field echo technique [fractional anisotropy (FA) = 8°; TE = 3.2 ms; TR = 7 ms; inversion time (TI) = 900 ms] with an isotropic voxel size of 1 mm^3^. With the help of the voxel tissue segmentation tool incorporated into Gannet 3.0 software (49), percentages of white matter (WM), GM, and cerebrospinal fluid (CSF) were calculated for each voxel. The fraction of GM (fGM) contributing to the observed MRS signal was calculated as fGM = GM%/(GM% + WM%) and was inserted into the statistical model as a covariate.

The ProFit program also provides a Cramér–Rao lower bound (CRLB), a measure of the quality of the metabolite quantification for each metabolite (50). Metabolites with CRLBs above 20% were excluded from the statistical analysis.

### Statistical Analysis

Two-sided independent *t*-tests and two-sided asymptotic Pearson chi-square, at the 5% significance level, were performed comparing demographic data between patients and HC. In addition, each metabolite was entered as a dependent variable in a univariate Generalized Linear Model (GLM) with group as the fixed factor and age, sex, and fGM as covariates. Finally, we performed GLM analyses only at the OCD group to investigate eventual associations between metabolic values and clinical data covarying for sex, age, and fGM. Statistical analysis was performed using Python version 3.6, and Bonferroni correction was applied considering the number of comparisons/metabolites analyzed in the study: separate corrections were performed for the main outcomes (three comparisons) and for the secondary outcomes (seven comparisons). The same rule was applied for the GLM seeking to evaluate the clinical associations; however, once we had three scales (Y-BOCS, BDI, and BAI) for each metabolite, corrections were performed for nine comparisons regarding the main outcomes and 21 comparisons for the secondary ones.

## Results

### Demographic Group Comparisons

One hundred forty-five volunteers were scanned with the two-dimensional JPRESS ^1^H-MRS sequence. Forty-four participants were excluded due to excessive movement or bad quality spectroscopy data. The final sample consisted of 101 participants: 59 patients with OCD and 42 healthy controls. Descriptives of demographic and clinical characteristics of both groups can be seen in Table 1: groups did not differ in age, sex, or gray matter fraction (fGM) in the voxel. It is worth mentioning that the mean Y-BOCS score was 29.3, which indicates a moderate to severe level of symptomatology according to Storch et al. (51) criteria. Regarding previous treatments, 42 patients have failed at least one selective serotonin reuptake inhibitor (SSRI), and 34 have failed to improve symptomatology after cognitive behavior therapy (CBT).

**Table 1:** Clinical and demographic variables in patients with obsessive-compulsive disorder and healthy controls.

	**Patients**	**Controls**	***t*/*X*^2^**	***p*-value**
	**(*n* = 59)**	**(*n* = 42)**		
Age (SD)	35.1 (10.0)	33.4 (11.5)	0.78	0.435^b^
Sex, female (%)	37 (62.7)	23 (57.5)	0.80	0.427^b^
Y-BOCS (SD)	29.3 (6.7)	–	–	–
BDI (SD)	17.7 (10.1)	–	–	–
BAI (SD)	16.0 (9.9)	–	–	–
Medications SSRI or SRI^1^ (%)	37 (62.7)	–	–	–
Other antidepressants^2^ (%)	4 (5.1)	–	–	–
Benzodiazepines^3^ (%)	4 (5.1)	–	–	–
Neuroleptics^4^ (%)	12 (20.3)	–	–	–
Anticonvulsants^5^ (%)	1 (1.7)	–	–	–
Stimulants^6^ (%)	1 (1.7)	–	–	–
fGM (SD)	0.69 (0.07)	0.71 (0.09)	−1.20	0.234^a^

*Y-BOCS, Yale-Brown Obsessive–Compulsive Scale; BDI, Beck Depression Inventory; BAI, Beck Anxiety Inventory; fGM, fractioned gray matter*.

1 *Fluoxetine, fluvoxamine, paroxetine, sertraline, citalopram, escitalopram, clomipramine*.

2 *Mirtazapine, venlafaxine, duloxetine*.

3 *Clonazepam*.

4 *Olanzapine, ziprasidone, quetiapine, aripiprazole*.

5 *Gabapentin*.

6 *Ritalin*.

a *Student's t-test for two independent samples (patients and healthy controls)*.

b *Two-sided asymptotic significance, Pearson chi-square*.

### ^1^H-MRS Results

Mean metabolite ratios relative to Cr are listed in Table 2 for each group. Due to CRLB above 20%, it was necessary to exclude 10 subjects for GABA and Gln and 62 subjects for Lac evaluation. Mean CRLB were 10.66% for GABA, 2.73% for Glu, 0.68% for Cr, 13.08% for Gln, 0.65% for NAA, 4.88% for GSH, 0.80% for Cho, 14.75% for Lac, and 2.99% for mI.

**Table 2:** Ventromedial PFC metabolites levels in patients with obsessive-compulsive disorder and healthy controls.

	***N***	**OCD**	**HC**	**Group-effect GLM**	
		**Mean (SD)**	**Mean (SD)**	***z***	***p*-value**
**Primary outcomes**					
Glu/Cr	101	0.70 (0.36)	0.77 (0.37)	2.540	0.011
GABA/Cr	91	0.29 (0.19)	0.30 (0.20)	−1.015	0.310
Glu/GABA	91	3.71 (3.68)	4.58 (5.37)	0.353	0.724
**Secondary outcomes**					
Gln/Cr	91	0.29 (0.15)	0.27 (0.13)	−0.658	0.511
Glu/Gln	91	2.96 (1.87)	3.23 (1.92)	1.821	0.069
NAA/Cr	101	1.05 (0.17)	1.04 (0.12)	1.578	0.110
GSH/Cr	101	0.26 (0.09)	0.26 (0.09)	0.028	0.977
Cho/Cr	101	0.16 (0.02)	0.16 (0.02)	0.021	0.983
Lac/Cr	39	0.15 (0.15)	0.14 (0.13)	−0.206	0.837
mI/Cr	101	0.43 (0.13)	0.43 (0.13)	0.096	0.923

*Glu, glutamate; GABA, gamma aminobutyric acid; Gln, Glutamine; NAA, n-acetylaspartate; GSH, glutathione; Cho, choline; Lac, lactate; ml, myo-inositol, z, t-stats*.

### Generalized Linear Model

**Figure 2:**
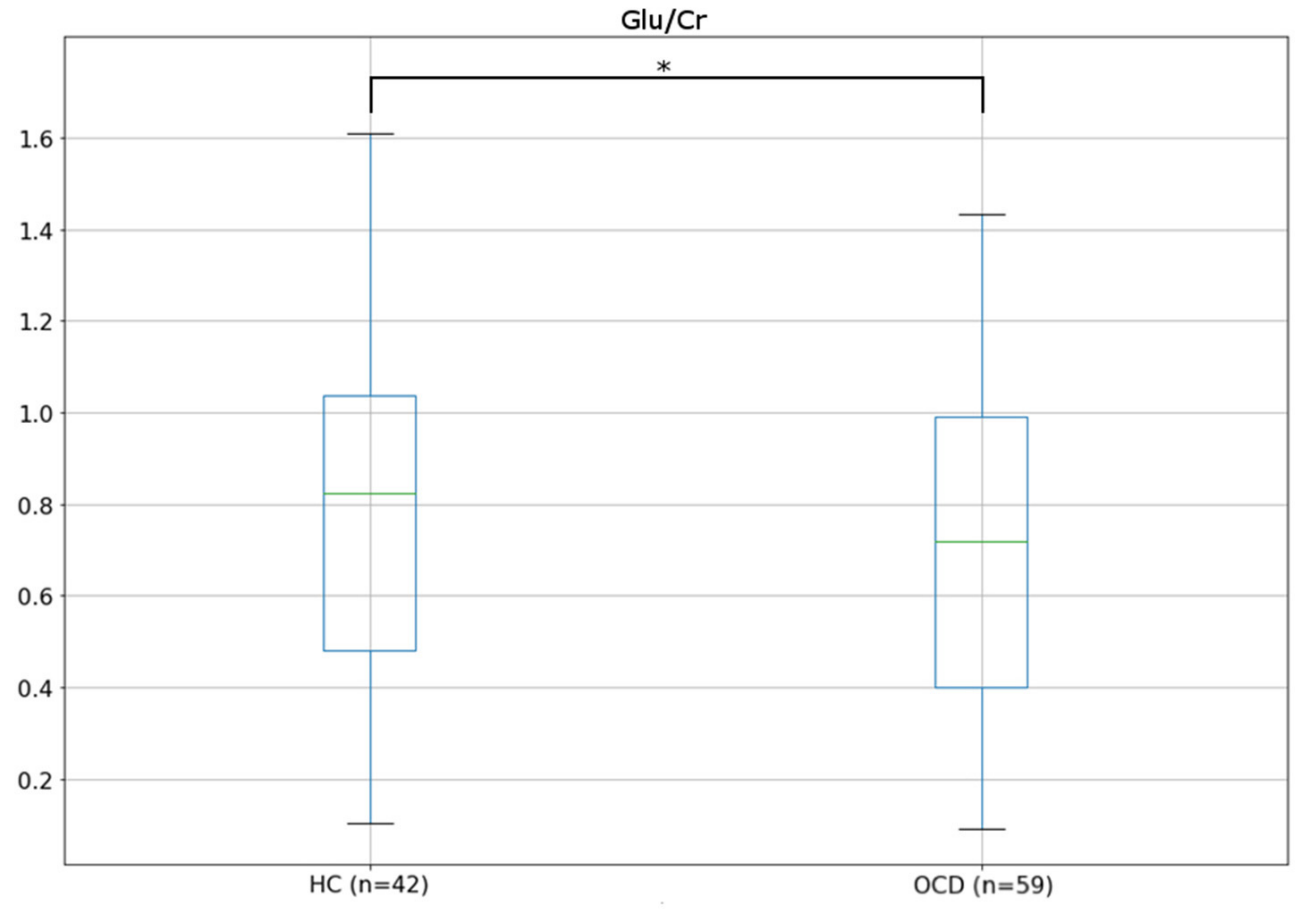
Boxplots showing the distribution of Glu/Cr in healthy controls (HCs) and patients with obsessive–compulsive disorder (OCD).

A summary of the group effect in the Generalized Linear Model (GLM) for each metabolite, together with the average for each group, is shown in Table 2. Multivariate regressions using GLM aimed to predict the variation in metabolites quantities using the following set of explanatory variables: (1) group, (2) sex, (3) age, and (4) fGM. We found a significant group effect for Glu/Cr, indicating that patients presented a lower concentration of this metabolite (*z* = 2.540; *p* = 0.011), a result that persisted after Bonferroni correction (Figure 2). On the other hand, GABA/Cr and the Glu/GABA ratio did not present any difference between groups (Table 2).

Regarding the secondary outcomes, none of the following models reached statistical significance or presented group effects: Gln/Cr, Glu/Gln, NAA/Cr, GSH/Cr, Cho/Cr, Lac/Cr, and mI/Cr (Table 2).

### Association With Clinical Symptoms

None of the clinical measures were associated with the metabolites for the primary or secondary outcomes: severity of OCD (Y-BOCS scores) and depressive or anxiety symptoms (BDI and BAI scores) (Supplementary Table 1).

## Discussion

The present study used a two-dimensional JPRESS ^1^H-MRS sequence to investigate specific metabolites in the ventromedial PFC (vmPFC) in one of the largest samples of patients with OCD reported to date. We found lower levels of Glu/Cr in patients compared with HC but no alterations in GABA/Cr or Glu/GABA ratio. We also did not observe any association of the metabolites with age or clinical symptoms nor group differences in the secondary outcomes.

Up to now, the literature regarding ^1^H-MRS of vmPFC in patients with OCD has presented inconclusive findings. Results are especially inconsistent when metabolites are reported as Glx: some studies reported higher levels of Glx in OCD compared with controls (8, 30), while others showed lower levels of Glx in patients (52). However, the vast majority of studies reported no differences (4, 6, 53). One explanation for the mixed findings could be related to the fact that most studies could not separate the spectral peaks of Glu and Gln, leading to ambiguity. Recent ^1^H-MRS techniques (as JPRESS) at high-field scanners (3T) are able to disentangle each metabolite concentration due to better spectral resolution, offering more precise results, i.e., Glu minimally contaminated by precursors and other metabolites.

Studies measuring Glu levels in adults with OCD and evaluating regions similar to ours have also found inconsistent results. While most of the literature found no differences in Glu values when comparing patients and controls (30, 53, 54), one study has found lower levels in the mPFC of OCD patients (29). Our results replicated, in a larger sample, the previous findings of Zhu et al. (29), who evaluated 13 patients with OCD. At first, these findings may seem contradictory, once the postulated hyperactivation of the CSTC would demand increased levels of Glu in frontal areas. However, it is noteworthy that no study using a 3T scan observed higher Glu values in the mPFC [for a review see Vester et al. (9)]. Moreover, the small sample size of previous studies (*n* = 16, 30, and 40) and the fact that only one study (53) used a JPRESS sequence could also account for the inconclusive results.

Lower levels of Glu in the vmPFC were also detected in children with OCD when compared to control youths (55, 56). While a straightforward comparison with these studies is difficult because of two main reasons, (1) a developing brain can be totally different in terms of functioning and neurochemistry from an adult brain and (2) both studies assessing children have used 1.5 T scans (and their samples were 20 and 14, respectively), it is possible that the brain mechanisms that underlie OCD symptoms could present similar pathophysiology, independently of age or developmental stage (57). In fact, our results support this view: even without having youth in our sample, we had participants with a wide age range. Although we tried to observe a pattern of association (reduction or increase) over the lifespan, we could not find age influences in any metabolite. In addition, this was a cross-sectional design study, which is not appropriate for this type of investigation.

The Glu alterations mentioned above and reported in our results may underlie the abnormalities in the frontostriatal circuits detected in OCD patients. The imbalance in Glu concentrations, especially in this frontal region, may reflect changes in the excitatory/inhibitory neurotransmission of the frontostriatal circuits, crucial in OCD pathophysiology, or even a compensatory mechanism. Finding similar results to ours, Rosenberg and colleagues (55), speculated that the pathophysiological model of OCD was associated with tonic–phasic dysregulation of Glu in CSTC circuits. Translating this hypothesis to our results, reduced tonic Glu levels in vmPFC could predispose to phasic Glu hyperactivity in the striatum and OFC. Although we did not measure metabolites concentrations in other areas, future studies should assess more than one voxel to have a greater view of the neurochemistry balance behind the CSTC circuitry in OCD patients. The clinical implication of a better understanding of the neurobiological model of the disease is relevant and useful. For example, a disrupted excitatory or inhibitory system in the vmPFC could be related to dysregulations in affective (26, 28) and cognitive systems (58), as reported in patients with OCD.

Overall, the lower levels in Glu/Cr presented by OCD patients support the glutamatergic hypothesis for OCD (10). The evidence for glutamatergic dysregulation in OCD has been increasingly strong in the literature (22), with alterations being detected in studies that analyzed neurochemical levels in the cerebrospinal fluid of unmedicated patients (59, 60) and also in ^1^H-MRS studies (29), as previously mentioned. Therefore, even with most ^1^H-MRS studies presenting negative results regarding Glu in several regions of the brain, we remember once more that most of the previous studies were underpowered and did not have a proper ^1^H-MRS sequence capable of separating Glu from Gln.

Regarding GABA/Cr, our findings are in line with other studies reporting no differences in vmPFC (5), including more recent studies that have used newer ^1^H-MRS sequences capable of measuring those metabolites more precisely (9). Although part of the literature emphasizes GABAergic abnormalities in the vmPFC of patients with OCD, with two studies reporting lower levels (4, 6) and a more recent one reporting higher levels (7) of this metabolite, we could not find any difference between groups. In fact, looking at the means for each group, they were impressively similar. Comparing the studies, our sample was larger than two of the previous studies (4, 7), and although the study of Zhang et al. (6) had a larger sample than ours, the voxel position was not exactly in the same region: while they preferred to evaluate the orbitofrontal cortex, in our study, we positioned the voxel in a more superior location, delimited by a straight line between the anterior and posterior commissures. These factors may have influenced and diverged our results from the previous studies.

Our JPRESS sequence allowed us to measure the Glu/Gln ratio, differently from previous studies that mainly reported only Glx (4, 6, 29, 30). Although we could not find differences between patients with OCD and HC, this ratio is particularly informative and was previously shown to be associated with other psychiatric disorders, like depression (61), autistic traits (62), and schizophrenia (63). Therefore, new ^1^H-MRS studies using pulse sequences that can distinguish Glu signals from Gln in OCD are warranted, not only to study their ratio but also to have a more precise and clear measure of each metabolite.

Regarding the secondary outcomes, our results also extend previous investigations of the involvement of these metabolites in the physiopathology of OCD: except for some studies that reported lower concentrations of NAA in the PFC of OCD patients (6, 64), most studies reported no alterations of Gln, NAA, GSH, Cho, Lac, or mI in the ventromedial PFC (29–31, 54, 65). Although this region is thought to be one of the most important of the cortico-striatal circuits involved with OCD symptoms, it seems that the evidence so far is insufficient to demonstrate alterations in other metabolites except Glu or GABA. Nevertheless, future studies with larger samples should investigate the longitudinal role of specific metabolites in OCD, whether they could be valid predictors of treatment response or if they could play a role in OCD symptoms improvement, as suggested in a recent umbrella review of biomarkers in OCD (58).

Even though we have found group effects in the Glu/Cr in this sample, none of the metabolites correlated with clinical symptoms, as measured by the Y-BOCS, BAI, or BDI, after a strict multiple comparisons correction, indicating that their levels are independent of clinical presentation or severity. Before correcting for multiple comparisons, GABA/Cr was associated with the severity of OCD symptoms and Glu/GABA with depression scores (regarding the mains outcomes), while Cho was associated with OCD and anxiety symptoms (secondary outcomes). With a sample of 59 patients with OCD, we cannot say that we were underpowered to observe correlations. On the other hand, the elevated number of comparisons does not allow us to interpret results without multiple comparison corrections. The literature offers mixed findings in this regard, with studies showing both positive (52) and negative (6) correlations of Glu (or Glx) and GABA in the vmPFC. However, previous studies did not control for multiple comparisons, and most ^1^H-MRS studies in the OCD literature did not find metabolic–clinical associations (4, 7, 54, 65). Therefore, it is difficult to establish a pattern, as concluded by a recent review of the literature (9), but our results reinforce these negative findings.

Limitations of this study should be observed. First, we did not exclude patients with comorbid depression. On the other hand, BDI scores definitely differed between groups and were not associated with metabolites levels, suggesting that depressive symptoms were independent of those measures. Second, although most of our patients were medicated at the time of the scan, we admitted patients taking medications only if they were stable for at least 6 weeks. Nevertheless, this could have influenced our findings. Thus, we ran analyses without patients taking anticonvulsants and benzodiazepines, and the results remained the same. Finally, we used a unique and non-commercial pulse sequence of MRS that could separate Glu/Cr and Gln/Cr, which strengthens our study. However, this was a very long sequence (lasting ~25 min), and thus, there was a higher chance that some subjects needed to be excluded from the analysis, simply because they moved their heads during this very long scanning. In addition, the voxel size was relatively large and positioned at the midline (as opposed to bilateral), encompassing diverse cortical subregions. Inevitably, the voxel contained also some white matter, which could affect results. In order to control for that, we verified that white matter portion was equal for both groups, and the gray matter fraction was considered in the statistical analysis.

To conclude, we found a group effect in the Glu/Cr concentration, which may indicate a neurochemical imbalance in the vmPFC of patients with OCD that could be associated with the corticostriatal dysregulation consistently implicated in the neurobiology of this disorder. It is hard to establish if the lower Glu/Cr levels found in patients with OCD in this study could be the cause or consequence of the disease or even related to any other pathophysiological process. They also could be the result of a compensatory system, as hypothesized before. Moreover, clinical severity of OCD, depression, and anxiety symptoms did not associate with metabolites levels, replicating previous studies. Our study sheds light on the relevance of further studying the glutamatergic system for understanding the neurobiology of the disease, for example, studies designed to test the predictive value of this metabolite and if/how it is affected by first-line treatments. It is possible that different OCD subtypes or patients that exhibit different clinical profiles would present different metabolite levels in the brain. Finally, future studies should evaluate other areas of the brain, particularly related to the CSTC circuits in patients with OCD.

### Conflicts of Interest

All the authors declare that the research was conducted in the absence of any commercial or financial relationships that could be construed as a potential conflict of interest. Dr. Batistuzzo confirms he had full access to all the data in the study and takes responsibility for the integrity of the data and the accuracy of the data analysis.

## Data Availability Statement

The raw data supporting the conclusions of this article will be made available by the authors, without undue reservation.

## Ethics Statement

This study, involving human participants, was reviewed and approved by Ethics Committee for Analysis of Research Projects (CAPPesq) board, of the Faculdade de Medicina da Universidade de São Paulo (FMUSP). The patients/participants provided their written informed consent to participate in this study.

## Author Contributions

MB planned the study, acquired and analyzed the data, wrote, and reviewed the manuscript. BS analyzed the data, wrote, and reviewed the manuscript. RGS and EM reviewed the manuscript. AL, CC, AM, JD, and RS acquired the data and reviewed the manuscript. BP helped to analyze the data and reviewed the manuscript. MH planned the study, wrote, and reviewed the manuscript. MO planned the study, analyzed the data, wrote, and reviewed the manuscript. All authors contributed to the article and approved the submitted version.

## Conflict of Interest

The authors declare that the research was conducted in the absence of any commercial or financial relationships that could be construed as a potential conflict of interest. The reviewer JO'N declared a shared consortium with several of the authors, MB, MH, JD, and EM, at time of review.

## References

[1] Ruscio AM Stein DJ Chiu WT Kessler RC. The epidemiology of obsessive-compulsive disorder in the National Comorbidity Survey Replication. Mol Psychiatry. (2010) 15:53–63. 10.1038/mp.2008.94 18725912 PMC2797569

[2] Andrade LH Wang YP Andreoni S Silveira CM Alexandrino-Silva C Siu ER. Mental disorders in megacities: findings from the São Paulo megacity mental health survey, Brazil. PLoS ONE. (2012) 7:e31879. 10.1371/journal.pone.0031879 22348135 PMC3279422

[3] Aoki Y Aoki A Suwa H. Reduction of N-acetylaspartate in the medial prefrontal cortex correlated with symptom severity in obsessive-compulsive disorder: meta-analyses of (1)H-MRS studies. Transl Psychiatry. (2012) 2:e153. 10.1038/tp.2012.78 22892718 PMC3432192

[4] Simpson HB Shungu DC Bender J Mao X Xu X Slifstein M. Investigation of cortical glutamate-glutamine and γ-aminobutyric acid in obsessive-compulsive disorder by proton magnetic resonance spectroscopy. Neuropsychopharmacology. (2012) 37:2684–92. 10.1038/npp.2012.132 22850733 PMC3473334

[5] Brennan BP Rauch SL Jensen JE Pope HG. A critical review of magnetic resonance spectroscopy studies of obsessive-compulsive disorder. Biol Psychiatry. (2013) 73:24–31. 10.1016/j.biopsych.2012.06.023 22831979 PMC3504626

[6] Zhang Z Fan Q Bai Y Wang Z Zhang H Xiao Z. Brain Gamma-Aminobutyric Acid (GABA) concentration of the prefrontal lobe in unmedicated patients with obsessive-compulsive disorder: a research of magnetic resonance spectroscopy. Shanghai Arch Psychiatry. (2016) 28:263–70. 10.11919/j.issn.1002-0829.216043 28638200 PMC5434282

[7] Li Y Zhang CC Kathrin Weidacker Zhang Y He N Jin H. Investigation of anterior cingulate cortex gamma-aminobutyric acid and glutamate-glutamine levels in obsessive-compulsive disorder using magnetic resonance spectroscopy. BMC Psychiatry. (2019) 19:164. 10.1186/s12888-019-2160-1 31146727 PMC6543571

[8] de Salles Andrade JB Ferreira FM Suo C Yücel M Frydman I Monteiro M. An MRI study of the metabolic and structural abnormalities in obsessive-compulsive disorder. Front Hum Neurosci. (2019) 13:186. 10.3389/fnhum.2019.00186 31333428 PMC6620433

[9] Vester EL de Joode NT Vriend C Pouwels PJW van den Heuvel OA. Little evidence for neurometabolite alterations in obsessive-compulsive disorder - a systematic review of magnetic resonance spectroscopy studies at 3 Tesla. J Obsessive-Compulsive Relat Disord. (2020) 25:100523. 10.1016/j.jocrd.2020.100523

[10] Pittenger C Bloch MH Williams K. Glutamate abnormalities in obsessive compulsive disorder: neurobiology, pathophysiology, and treatment. Pharmacol Ther. (2011) 132:314–32. 10.1016/j.pharmthera.2011.09.006 21963369 PMC3205262

[11] Wu K Hanna GL Rosenberg DR Arnold PD. The role of glutamate signaling in the pathogenesis and treatment of obsessive-compulsive disorder. Pharmacol Biochem Behav. (2012) 100:726–35. 10.1016/j.pbb.2011.10.007 22024159 PMC3437220

[12] Stewart SE Mayerfeld C Arnold PD Crane JR O'Dushlaine C Fagerness JA. Meta-analysis of association between obsessive-compulsive disorder and the 3' region of neuronal glutamate transporter gene SLC1A1. Am J Med Genet B Neuropsychiatr Genet. (2013) 162B:367–79. 10.1002/ajmg.b.32137 23606572

[13] Marinova Z Chuang DM Fineberg N. Glutamate-modulating drugs as a potential therapeutic strategy in obsessive-compulsive disorder. Curr Neuropharmacol. (2017) 15:977–95. 10.2174/1570159X15666170320104237 28322166 PMC5652017

[14] Karthik S Sharma LP Narayanaswamy JC. Investigating the role of glutamate in obsessive-compulsive disorder: current perspectives. Neuropsychiatr Dis Treat. (2020) 16:1003–13. 10.2147/NDT.S211703 32368062 PMC7173854

[15] Rodriguez CI Kegeles LS Levinson A Feng T Marcus SM Vermes D. Randomized controlled crossover trial of ketamine in obsessive-compulsive disorder: proof-of-concept. Neuropsychopharmacology. (2013) 38:2475–83. 10.1038/npp.2013.150 23783065 PMC3799067

[16] Rodriguez CI Kegeles LS Levinson A Ogden RT Mao X Milak MS. *In vivo* effects of ketamine on glutamate-glutamine and gamma-aminobutyric acid in obsessive-compulsive disorder: proof of concept. Psychiatry Res. (2015) 233:141–7. 10.1016/j.pscychresns.2015.06.001 26104826 PMC4715460

[17] Oliver G Dean O Camfield D Blair-West S Ng C Berk M. N-acetyl cysteine in the treatment of obsessive compulsive and related disorders: a systematic review. Clin Psychopharmacol Neurosci. (2015) 13:12–24. 10.9758/cpn.2015.13.1.12 25912534 PMC4423164

[18] Li F Welling MC Johnson JA Coughlin C Mulqueen J Jakubovski E. N-Acetylcysteine for pediatric obsessive-compulsive disorder: a small pilot study. J Child Adolesc Psychopharmacol. (2020) 30:32–7. 10.1089/cap.2019.0041 31800306 PMC7133418

[19] Costa DLC Diniz JB Requena G Joaquim MA Pittenger C Bloch MH. Randomized, double-blind, placebo-controlled trial of N-acetylcysteine augmentation for treatment-resistant obsessive-compulsive disorder. J Clin Psychiatry. (2017) 78:e766–73. 10.4088/JCP.16m11101 28617566

[20] Sheshachala K Narayanaswamy JC. Glutamatergic augmentation strategies in obsessive-compulsive disorder. Indian J Psychiatry. (2019) 61(Suppl 1):S58–65. 10.4103/psychiatry.IndianJPsychiatry_520_18 30745678 PMC6343415

[21] International Obsessive-Compulsive Disorder Foundation Genetics Collaborative (IOCDF-GC) and OCD Collaborative Genetics Association Studies (OCGAS). Revealing the complex genetic architecture of obsessive-compulsive disorder using meta-analysis. Mol Psychiatry. (2018) 23:1181–8. 10.1038/mp.2017.154 28761083 PMC6660151

[22] Stein DJ Costa DLC Lochner C Miguel EC Reddy YCJ Shavitt RG. Obsessive-compulsive disorder. Nat Rev Dis Primers. (2019) 5:52. 10.1038/s41572-019-0102-3 31371720 PMC7370844

[23] Shephard E Stern ER van den Heuvel OA Costa DLC Batistuzzo MC Godoy PBG. Toward a neurocircuit-based taxonomy to guide treatment of obsessive-compulsive disorder. Mol Psychiatry. (2021). 10.1038/s41380-020-01007-8. [Epub ahead of print]. 33414496 PMC8260628

[24] Dougherty DD Brennan BP Stewart SE Wilhelm S Widge AS Rauch SL. Neuroscientifically informed formulation and treatment planning for patients with obsessive-compulsive disorder: a review. JAMA Psychiatry. (2018) 75:1081–7. 10.1001/jamapsychiatry.2018.0930 30140845

[25] Graybiel AM Rauch SL. Toward a neurobiology of obsessive-compulsive disorder. Neuron. (2000) 28:343–7. 10.1016/S0896-6273(00)00113-6 11144344

[26] Milad MR Rauch SL. Obsessive-compulsive disorder: beyond segregated cortico-striatal pathways. Trends Cogn Sci. (2012) 16:43–51. 10.1016/j.tics.2011.11.003 22138231 PMC4955838

[27] van den Heuvel OA Remijnse PL Mataix-Cols D Vrenken H Groenewegen HJ Uylings HB. The major symptom dimensions of obsessive-compulsive disorder are mediated by partially distinct neural systems. Brain. (2009) 132:853–68. 10.1093/brain/awn267 18952675

[28] Milad MR Rosenbaum BL Simon NM. Neuroscience of fear extinction: implications for assessment and treatment of fear-based and anxiety related disorders. Behav Res Ther. (2014) 62:17–23. 10.1016/j.brat.2014.08.006 25204715

[29] Zhu Y Fan Q Han X Zhang H Chen J Wang Z. Decreased thalamic glutamate level in unmedicated adult obsessive-compulsive disorder patients detected by proton magnetic resonance spectroscopy. J Affect Disord. (2015) 178:193–200. 10.1016/j.jad.2015.03.008 25819113

[30] O'Neill J Lai TM Sheen C Salgari GC Ly R Armstrong C. Cingulate and thalamic metabolites in obsessive-compulsive disorder. Psychiatry Res Neuroimaging. (2016) 254:34–40. 10.1016/j.pscychresns.2016.05.005 27317876 PMC5780184

[31] Yücel M Harrison BJ Wood SJ Fornito A Wellard RM Pujol J. Functional and biochemical alterations of the medial frontal cortex in obsessive-compulsive disorder. Arch Gen Psychiatry. (2007) 64:946–55. 10.1001/archpsyc.64.8.946 17679639

[32] Rajendram R Kronenberg S Burton CL Arnold PD. Glutamate genetics in obsessive-compulsive disorder: a review. J Can Acad Child Adolesc Psychiatry. (2017) 26:205–13. 29056983 PMC5642460

[33] Schulte RF Lange T Beck J Meier D Boesiger P. Improved two-dimensional J-resolved spectroscopy. NMR Biomed. (2006) 19:264–70. 10.1002/nbm.1027 16541465

[34] Schulte RF Boesiger P. ProFit: two-dimensional prior-knowledge fitting of J-resolved spectra. NMR Biomed. (2006) 19:255–63. 10.1002/nbm.1026 16541464

[35] Scotti-Muzzi E Chile T Moreno R Pastorello BF da Costa Leite C Henning A. ACC Glu/GABA ratio is decreased in euthymic bipolar disorder I patients: possible *in vivo* neurometabolite explanation for mood stabilization. Eur Arch Psychiatry Clin Neurosci. (2020) 271:537–47. 10.1007/s00406-020-01096-0 31993746

[36] First MB Spitzer RL Gibbon M Williams JBW. Structured Clinical Interview for DSM-IV Axis I Disorders. Patient Edition (SCID-I/P). New York, NY: Biometrics Research, New York State Psychiatric Institute (1998).

[37] Gorenstein C Andrade LHSG Zuardi AW. Escalas de avaliação clínica em psiquiatria e psicofarmacologia. São Paulo: Lemos Editorial (2000).

[38] Goodman WK Price LH Rasmussen SA Mazure C Fleischmann RL Hill CL. The yale-brown obsessive compulsive scale. I. development, use, and reliability. Arch Gen Psychiatry. (1989) 46:1006–11. 10.1001/archpsyc.1989.01810110048007 2684084

[39] Fatori D Costa DL Asbahr FR Ferrão YA Rosário MC Miguel EC. Is it time to change the gold standard of obsessive-compulsive disorder severity assessment? Factor structure of the Yale-Brown Obsessive-Compulsive Scale. Aust N Z J Psychiatry. (2020) 54:732–42. 10.1177/0004867420924113 32475123

[40] Beck AT Ward CH Mendelson M Mock J Erbaugh J. An inventory for measuring depression. Arch Gen Psychiatry. (1961) 4:561–71. 10.1001/archpsyc.1961.01710120031004 13688369

[41] Beck AT Epstein N Brown G Steer RA. An inventory for measuring clinical anxiety: psychometric properties. J Consult Clin Psychol. (1988) 56:893–7. 10.1037/0022-006X.56.6.893 3204199

[42] Cunha JA. Manual da versão em português das Escalas Beck. São Paulo: Casa do Psicólogo (2001).

[43] Tkác I Starcuk Z Choi IY Gruetter R. *In vivo* 1H NMR spectroscopy of rat brain at 1 ms echo time. Magn Reson Med. (1999) 41:649–56.3. 10.1002/(SICI)1522-2594(199904)41:4<649::AID-MRM2>3.0.CO;2-G 10332839

[44] Fuchs A Boesiger P Schulte RF Henning A. ProFit revisited. Magn Reson Med. (2014) 71:458–68. 10.1002/mrm.24703 23475809

[45] Provencher SW. Estimation of metabolite concentrations from localized *in vivo* proton NMR spectra. Magn Reson Med. (1993) 30:672–9. 10.1002/mrm.1910300604 8139448

[46] Smith SA Levante TO de Beer R Luyten PR van Ormondt D. Computer simulations in magnetic resonance. an object-oriented programming approach. J Magnet Reson. (1994) 106:75–105. 10.1006/jmra.1994.1008

[47] Fan TWM. Metabolite profiling by one- and two-dimensional NMR analysis of complex mixtures. Prog Nuclear Magnet Reson Spectroscop. (1996) 28:161–219. 10.1016/0079-6565(96)90002-3

[48] Govindaraju V Young K Maudsley AA. Proton NMR chemical shifts and coupling constants for brain metabolites. NMR Biomed. (2000) 13:129–53. 10.1002/1099-1492(200005)13:3<129::AID-NBM619>3.0.CO;2-V 10861994

[49] Edden RA Puts NA Harris AD Barker PB Evans CJ. Gannet: a batch-processing tool for the quantitative analysis of gamma-aminobutyric acid–edited MR spectroscopy spectra. J Magn Reson Imaging. (2014) 40:1445–52. 10.1002/jmri.24478 25548816 PMC4280680

[50] Cavassila S Deval S Huegen C van Ormondt D Graveron-Demilly D. Cramér-Rao bounds: an evaluation tool for quantitation. NMR Biomed. (2001) 14:278–83. 10.1002/nbm.701 11410946

[51] Storch EA De Nadai AS Conceição do Rosário M Shavitt RG Torres AR Ferrão YA. Defining clinical severity in adults with obsessive-compulsive disorder. Compr Psychiatry. (2015) 63:30–5. 10.1016/j.comppsych.2015.08.007 26555489 PMC4643407

[52] Yücel M Wood SJ Wellard RM Harrison BJ Fornito A Pujol J. Anterior cingulate glutamate-glutamine levels predict symptom severity in women with obsessive-compulsive disorder. Aust N Z J Psychiatry. (2008) 42:467–77. 10.1080/00048670802050546 18465373

[53] Brennan BP Tkachenko O Schwab ZJ Juelich RJ Ryan EM Athey AJ. An examination of rostral anterior cingulate cortex function and neurochemistry in obsessive-compulsive disorder. Neuropsychopharmacology. (2015) 40:1866–76. 10.1038/npp.2015.36 25662837 PMC4839510

[54] Zurowski B Kordon A Weber-Fahr W Voderholzer U Kuelz AK Freyer T. Relevance of orbitofrontal neurochemistry for the outcome of cognitive-behavioural therapy in patients with obsessive-compulsive disorder. Eur Arch Psychiatry Clin Neurosci. (2012) 262:617–24. 10.1007/s00406-012-0304-0 22427151

[55] Rosenberg DR Mirza Y Russell A Tang J Smith JM Banerjee SP. Reduced anterior cingulate glutamatergic concentrations in childhood OCD and major depression versus healthy controls. J Am Acad Child Adolesc Psychiatry. (2004) 43:1146–53. 10.1097/01.chi.0000132812.44664.2d 15322418

[56] Arnold PD Macmaster FP Richter MA Hanna GL Sicard T Burroughs E. Glutamate receptor gene (GRIN2B) associated with reduced anterior cingulate glutamatergic concentration in pediatric obsessive-compulsive disorder. Psychiatry Res. (2009) 172:136–9. 10.1016/j.pscychresns.2009.02.005 19324536 PMC2670773

[57] Kalra SK Swedo SE. Children with obsessive-compulsive disorder: are they just “little adults”? J Clin Invest. (2009) 119:737–46. 10.1172/JCI37563 19339765 PMC2662563

[58] Fullana MA Abramovitch A Via E López-Sola C Goldberg X Reina N. Diagnostic biomarkers for obsessive-compulsive disorder: a reasonable quest or ignis fatuus? Neurosci Biobehav Rev. (2020) 118:504–13. 10.1016/j.neubiorev.2020.08.008 32866526

[59] Chakrabarty K Bhattacharyya S Christopher R Khanna S. Glutamatergic dysfunction in OCD. Neuropsychopharmacology. (2005) 30:1735–40. 10.1038/sj.npp.1300733 15841109

[60] Bhattacharyya S Khanna S Chakrabarty K Mahadevan A Christopher R Shankar SK. Anti-brain autoantibodies and altered excitatory neurotransmitters in obsessive-compulsive disorder. Neuropsychopharmacology. (2009) 34:2489–96. 10.1038/npp.2009.77 19675532

[61] Yüksel C Öngür D. Magnetic resonance spectroscopy studies of glutamate-related abnormalities in mood disorders. Biol Psychiatry. (2010) 68:785–94. 10.1016/j.biopsych.2010.06.016 20728076 PMC2955841

[62] Kondo HM Lin IF. Excitation-inhibition balance and auditory multistable perception are correlated with autistic traits and schizotypy in a non-clinical population. Sci Rep. (2020) 10:8171. 10.1038/s41598-020-65126-6 32424307 PMC7234986

[63] Bustillo JR Chen H Jones T Lemke N Abbott C Qualls C. Increased glutamine in patients undergoing long-term treatment for schizophrenia: a proton magnetic resonance spectroscopy study at 3 T. JAMA Psychiatry. (2014) 71:265–72. 10.1001/jamapsychiatry.2013.3939 24402128 PMC8185982

[64] Tükel R Aydin K Ertekin E Özyildirim S Taravari V. Proton magnetic resonance spectroscopy in obsessive-compulsive disorder: evidence for reduced neuronal integrity in the anterior cingulate. Psychiatry Res. (2014) 224:275–80. 10.1016/j.pscychresns.2014.08.012 25241042

[65] Hatchondo L Jaafari N Langbour N Maillochaud S Herpe G Guillevin R. H magnetic resonance spectroscopy suggests neural membrane alteration in specific regions involved in obsessive-compulsive disorder. Psychiatry Res Neuroimaging. (2017) 269:48–53. 10.1016/j.pscychresns.2017.08.010 28938221

