# Supplementary material for: Lower Ventromedial Prefrontal Cortex Glutamate Levels in Patients With Obsessive–Compulsive Disorder
Source: Front Psychiatry. 2021 Jun 8;12:668304. doi: 10.3389/fpsyt.2021.668304 (PMC8218991; doi:10.3389/fpsyt.2021.668304)
Supplement: Supplementary file 1 [file Image_1.pdf]

Supplementary Material

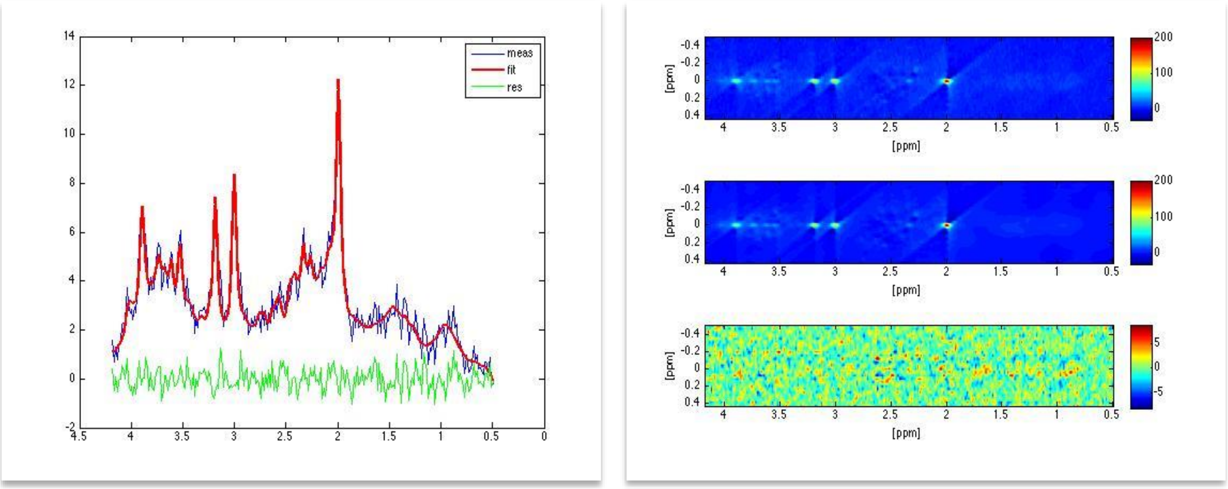

Supplementary Figure 1. An example of a volunteer's ^{1}H-MRS JPRESS spectrum after being processed by ProFit 2.0. On the left side, the green line represents the residuals, the blue line is the real spectrum and the red line is the fitted model. The horizontal axis represents ppm and the vertical axis arbitrary units representing the signal intensity; on the right: the top row represents the original 2D spectrum, the middle row represents the fit to the spectrum, and at the bottom, the residual of the fit. Note that the intensity scale of the residual is magnified.
