# Supplementary material for: Lower Ventromedial Prefrontal Cortex Glutamate Levels in Patients With Obsessive–Compulsive Disorder
Source: Front Psychiatry. 2021 Jun 8;12:668304. doi: 10.3389/fpsyt.2021.668304 (PMC8218991; doi:10.3389/fpsyt.2021.668304)
Supplement: Supplementary file 2 [file Table_1.pdf]

Supplementary Material

Supplementary Table 1. Association between metabolites and clinical symptoms in our sample of patients with OCD (n = 59).

	**Y-BOCS**		**BAI**		**BDI**	
	**z**	**p-value***	**z**	**p-value***	**z**	**p-value***
**Primary outcomes**						
**Glu/Cr**	-0.459	0.646	-0.973	0.331	0.794	0.427
**GABA/Cr**	2.066	0.039	-0.701	0.483	-1.476	0.140
**Glu/GABA**	-1.806	0.071	-0.424	0.672	2.461	0.014
**Secondary outcomes**						
**Gln/Cr**	0.787	0.431	-0.176	0.860	-0.498	0.618
**Glu/Gln**	-1.924	0.054	-0.289	0.772	0.965	0.335
**NAA/Cr**	-0.292	0.770	-0.990	0.368	-0.221	0.825
**GSH/Cr**	0.701	0.484	0.042	0.967	-1.101	0.271
**Cho/Cr**	2.377	0.017	-1.823	0.068	-0.344	0.730
**Lac/Cr**	0.385	0.700	-0.743	0.458	-0.642	0.521
**mI/Cr**	-0.402	0.687	0.20	0.834	0.713	0.476

Glu: glutamate; GABA: gamma aminobutyric acid; Gln: Glutamine; NAA: n-acetylaspartate; GSH: glutathione; Cho: choline; Lac: lactate; ml: myo-inositol; Cr: Creatine. z: t-stats. * uncorrected p-value
